# Effect of milk replacer allowance on calf faecal bacterial community profiles and fermentation

**DOI:** 10.1186/s42523-021-00088-2

**Published:** 2021-04-01

**Authors:** Sandeep Kumar, M. Ajmal Khan, Emma Beijer, Jinxin Liu, Katherine K. Lowe, Wayne Young, David A. Mills, Christina D. Moon

**Affiliations:** 1 AgResearch Limited, Grasslands Research Centre, Palmerston North, New Zealand; 2 Animal Nutrition Group, Wageningen University and Research, Wageningen, The Netherlands; 3 Department of Food Science and Technology, Robert Mondavi Institute for Wine and Food Science, University of California, Davis, One Shields Ave, Davis, CA 95616 USA; 4 Foods for Health Institute, University of California, Davis, California, One Shields Ave, Davis, CA 95616 USA; 5 Department of Viticulture and Enology, Robert Mondavi Institute for Wine and Food Science, University of California, Davis, California, One Shields Ave, Davis, CA 95616 USA

**Keywords:** Bovine, Diet, Gut, Microbiota, Nutrition, Ruminant, Short-chain fatty acid

## Abstract

**Background:**

The nutrition of calves from birth until weaning is predominantly from liquid (milk or milk-based) feeds. Liquid feed allowances are often restricted during artificial rearing to accelerate the development of the rumen by promoting solid feed intake. Liquid feeds bypass the rumen and are digested in the lower digestive tract, however, the influence of different types of milk feeds, and their allowances, on the calf hindgut microbiota is not well understood. In this study, faecal samples from 199 calves raised on three different allowances of milk replacer: 10% of initial bodyweight (LA), 20% of initial bodyweight (HA), and ad libitum (ADLIB), were collected just prior to weaning. Bacterial community structures and fermentation products were analysed, and their relationships with calf growth and health parameters were examined to identify potential interactions between diet, gut microbiota and calf performance.

**Results:**

Differences in the total concentrations of short-chain fatty acids were not observed, but higher milk replacer allowances increased the concentrations of branched short-chain fatty acids and decreased acetate to propionate ratios. The bacterial communities were dominated by *Ruminococcaceae*, *Lachnospiraceae* and *Bacteroides,* and the bacterial diversity of the ADLIB diet group was greater than that of the other diet groups. *Faecalibacterium* was over three times more abundant in the ADLIB compared to the LA group, and its abundance correlated strongly with girth and body weight gains. Milk replacer intake correlated strongly with *Peptococcus* and *Blautia*, which also correlated with body weight gain. *Bifidobacterium* averaged less than 1% abundance, however its levels, and those of *Clostridium* sensu stricto 1, correlated strongly with initial serum protein levels, which are an indicator of colostrum intake and passive transfer of immunoglobulins in early life.

**Conclusions:**

Higher milk replacer intakes in calves increased hindgut bacterial diversity and resulted in bacterial communities and short chain fatty acid profiles associated with greater protein fermentation. Increased abundances of beneficial bacteria such as *Faecalibacterium,* were also observed, which may contribute to development and growth. Moreover, correlations between microbial taxa and initial serum protein levels suggest that colostrum intake in the first days of life may influence microbiota composition at pre-weaning.

**Supplementary Information:**

The online version contains supplementary material available at 10.1186/s42523-021-00088-2.

## Background

Mature ruminants derive the majority of their energy requirements from the end products of rumen fermentation, short-chain fatty acids (SCFA), produced during the digestion of feed. However, ruminants are born with under-developed rumens and from birth until weaning, are highly reliant on milk-based feeds which bypass the rumen [1] and are largely digested in the lower gut. Gastrointestinal disorders of the lower gut are common in young ruminants and a leading cause of morbidity and mortality of dairy calves [2]. Thus, the microbial communities of the lower gut are particularly important for pre-weaned ruminants as they contribute to nutrition, gut development and homeostasis. Moreover, it is increasingly recognised that the influence of the intestinal microbiota extends beyond these activities, contributing also to detoxification, immune system development, behaviour, among other factors, thus having a broader influence on growth, development, health and wellbeing [3].

Characterisation of the calf intestinal microbiota is generally undertaken through the analysis of faecal samples [4–7], though the characterisation of microbial communities along the gastrointestinal tract is also commonly performed, which allows a comprehensive view of the development of both foregut and hindgut communities in concert [8–10]. Diverse intestinal microbiota have been detected within 30 min of birth, where *Proteobacteria* comprised over 30% of 16S rRNA gene sequences [7]. Over the following weeks, the community becomes dominated by characteristic gut anaerobic phyla, *Firmicutes* and *Bacteroidetes* [5, 7]. Diet has a large impact on gut microbiota diversity [3, 11], but our understanding of how different feeding practises used for calf rearing impact the intestinal microbiota and function is relatively limited. The inclusion of calf starter to a milk replacer diet increased the species richness of intestinal microbiota in 49 day old calves [10], and differences in the bacterial and archaeal intestinal communities were observed between calves fed corn silage compared to calves receiving concentrate-based starter diets [4]. Higher allowances of whole milk to calves promoted the abundance of *Faecalibacterium*, a butyrate producer and an important gut commensal in healthy animals, and was associated with greater concentrations of caecal butyrate (Moon et al. unpubl. observation), which is used by gut epithelial cells and contributes to gut development and homeostasis. *Faecalibacterium* were also dominant in the calf caecum and colon microbiota of 7-week old calves [8], and were associated with greater weight gains and lower incidences of diarrhoea in calves [5], where their potential as a probiotic for calves is being explored [12, 13].

In New Zealand’s dairy production systems, it is common practice for calves to be collected from their dams within 24 h of birth and artificially reared in groups in the absence of the dam using whole milk or milk replacers. Moreover, allowances of milk feeds are often restricted to encourage greater intakes of solid feed (often grain-based calf starters) to promote rumen development and earlier weaning. An aim of calf feeding is to promote pre-weaning growth of calves because faster growth rates from higher allowances of milk feeds have been associated with greater future milk yield in dairy heifers [14]. There is increasing evidence that greater pre-weaning growth and future milk yield of dairy heifers could be attributed to the effects of milk feeds on the development of the mammary gland and gastrointestinal tract, including the gut microbiome [2, 15–17].

The present study builds upon the findings of Groenendijk et al. (2018) who evaluated the effect of different allowances (low, high and ad libitum) of milk replacer dispensed using automated milk feeders, on the performance of nearly 200 dairy heifer calves [18]. Calves that had ad libitum access to milk replacer had greater average daily gains compared to the high and low allowance treatments and were associated with enhanced development of the mammary gland during pre-weaning. Therefore, we sought to understand the relationships between the gut microbiota and hindgut fermentation with calf growth and health by characterising the faecal microbial communities and fermentation products. This study provides further insights into the influence of milk replacer allowance on the gut microbiota and their contributions to calf growth and development prior to weaning.

## Results

The faecal microbiomes of the calves from our previous study [18] were examined using faecal samples obtained just prior to weaning. The low allowance (LA) group were allowed milk replacer to a maximum of 10% (vol/wt) of their initial body weight per day; the high allowance (HA) group were allowed milk replacer to a maximum of 20% (vol/wt) of their initial body weight per day; and the ad libitum (ADLIB) group were given ad libitum access to milk replacer. All calves had ad libitum access to calf starter.

The total faecal SCFA concentrations did not differ among the treatment groups, but the branched short-chain fatty acids (BSCFA), isobutyric acid and isovaleric acid, were in higher concentrations in calves on the ADLIB treatment (ANOVA, *P* < 0.001; Table 1). When expressed as a percentage of the total SCFA concentration, all SCFAs differed significantly (ANOVA, *P* < 0.05; Table 1). Acetic acid levels decreased with increasing allowances of milk replacer, while propionic acid increased. Isobutyric, valeric and isovaleric acids all also increased with increasing allowances of milk replacer.

**Table 1:** Faecal SCFA measurements

VFAs	LA		HA		ADLIB		FDR^1^
	Mean	SEM^2^	Mean	SEM	Mean	SEM	
*Concentration (mM)*							
Total SCFA	57.55	3.96	56.24	3.37	57.98	4.80	0.421
Acetic acid	40.61	2.68	39.21	2.56	37.09	2.77	0.378
Propionic acid	9.29	0.81	9.58	0.66	11.71	1.27	0.336
Butyric acid	5.29	0.61	3.89	0.32	4.61	0.62	0.159
Valeric acid	0.75	0.06	0.97	0.07	0.90	0.10	0.104
Isobutyric acid	0.89^b^	0.08	1.42^a^	0.13	1.85^a^	0.26	< 0.001***
Isovaleric acid	0.76^b^	0.08	1.20^b^	0.12	1.81^a^	0.31	< 0.001***
Acetate/propionate	5.03^a^	0.44	4.20^ab^	0.13	3.46^b^	0.17	< 0.001***
*Proportion (%)*							
Acetic acid	71.40^a^	0.84	69.55^a^	0.80	65.12^b^	1.06	< 0.001***
Propionic acid	15.57^b^	0.57	16.92^b^	0.46	19.63^a^	0.65	< 0.001***
Butyric acid	8.54^a^	0.61	6.86^b^	0.30	7.33^ab^	0.39	0.0373*
Valeric acid	1.35^b^	0.10	1.76^a^	0.09	1.74^a^	0.16	< 0.001***
Isobutyric acid	1.73^b^	0.16	2.66^a^	0.24	3.22^a^	0.20	0.0193*
Isovaleric acid	1.47^c^	0.15	2.27^b^	0.22	3.02^a^	0.25	< 0.001***

^1^Permutational multivariate analysis of variance (PERMANOVA) was performed to test for differences between the LA (*n* = 33), HA (*n* = 26), and ADLIB (*n* = 28) treatment groups. *P*-values were adjusted using the false discovery rate (FDR) method. Asterisks indicated FDR significance at *P* < 0.05 (*) and *P* < 0.001 (***). For variables with FDR < 0.05, Fisher’s least significant difference test was performed and results are shown in superscript next to the mean values

^2^Standard error of the mean

### Effect of milk replacer allowance on faecal bacterial diversity

**Fig. 1:**
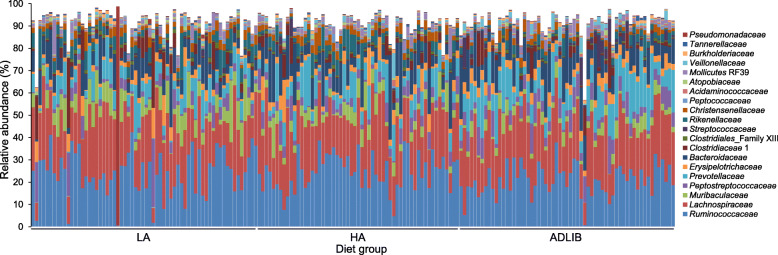
Barcharts of calf faecal microbiota compositions showing relative abundances of core family level taxa with an average abundance > 0.01% and present in > 90% of all samples. Samples are grouped by diet treatment

An average (± SEM) of 16,062 ± 490 high quality partial bacterial 16S rRNA gene sequence reads per sample were obtained after taxonomic assignment of reads using the SILVA V132 database [19] and omission of low read samples (< 3000 reads per sample). Overall, 35 bacterial families of > 0.01% relative abundance (Table S1; Fig. 1), and 108 genera of > 0.01% relative abundance (Table S2) were identified. *Ruminococcaceae*, *Lachnospiraceae*, *Bacteroidaceae*, *Prevotellaceae* and *Muribaculaceae* were the most dominant families across all treatment groups, together comprising over 60% of all reads on average, with mean relative abundances varying between 3.4 to 24.6% (Fig. 1). The relative abundances of the abundant families *Ruminococcaceae*, *Prevotellaceae* and *Peptostreptococcaceae* differed significantly between the treatment groups (Kruskal-Wallis test, *P* < 0.05), where *Ruminococcaceae* increased from 20.8 to 24.0%, *Prevotellaceae* decreased from 11.7 to 7.0%, and *Peptostreptococcaceae* decreased from 5.8 to 3.8% in mean relative abundance from the LA to the ADLIB groups (Table S1). Abundant bacterial families that exhibited relatively large and significant shifts in abundance (Kruskal-Wallis test, *P* < 0.05) between the diet groups included *Muribaculaceae* which increased from 3.5 to 6.8%, and *Rikenellaceae* which increased from 2.0 to 3.8% from the LA compared to the ADLIB treatment (Table S1).

Among abundant genera that appeared to be promoted by higher milk replacer allowances, the mean abundance of *Blautia* increased from 2.7 to 6.4%, *Alloprevotella* from 2.3 to 4.5% and *Faecalibacterium* from 0.8 to 2.6% in the ADLIB compared to the LA group. In contrast, taxa such as *Muribaculaceae* uncultured, *Rikenellaceae* RC9 gut group, *Ruminococcaceae* UCG 010 and *Roseburia* all decreased in abundance by at least 50% between these treatments (Table S2).

**Fig. 2:**
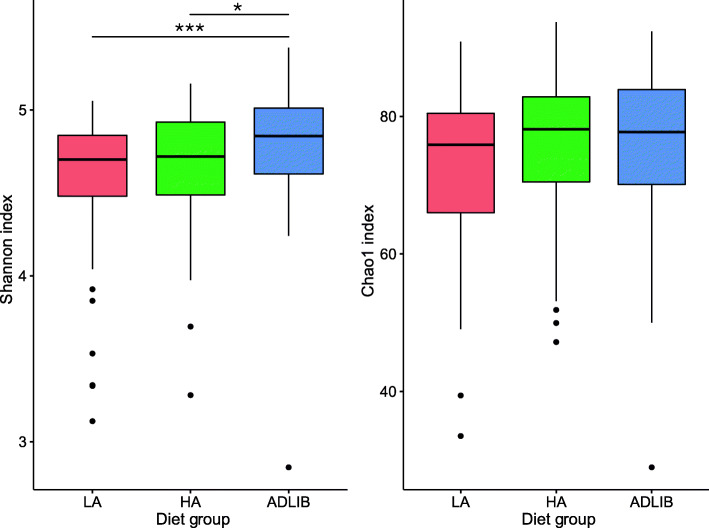
Boxplots showing alpha diversity distributions of calf faecal bacterial communities at the genus level by Shannon diversity index and Chao1 index between diet groups. The results of Wilcoxon rank sum testing of pairwise comparisons of treatments groups are shown for *P* < 0.05 (*), and *P* < 0.001 (**)

Alpha diversity was measured by both Shannon diversity index and Chao1 index to monitor bacterial community diversity at the genus level (Fig. 2). Kruskal-Wallis tests for differences in diversity indices between diet groups of all calves (*n* = 181) identified significant differences for Shannon diversity index (*P* = 0.0041), but not for Chao1 (*P* = 0.15). Pairwise comparisons of treatment groups showed that the difference in Shannon index data of the ADLIB treatment group differed significantly to those of the LA group (Wilcoxon rank sum test *P* < 0.001) and the HA group (*P* = 0.03) (Fig. 2).

### Effect of milk replacer allowance on faecal bacterial community structure

**Fig. 3:**
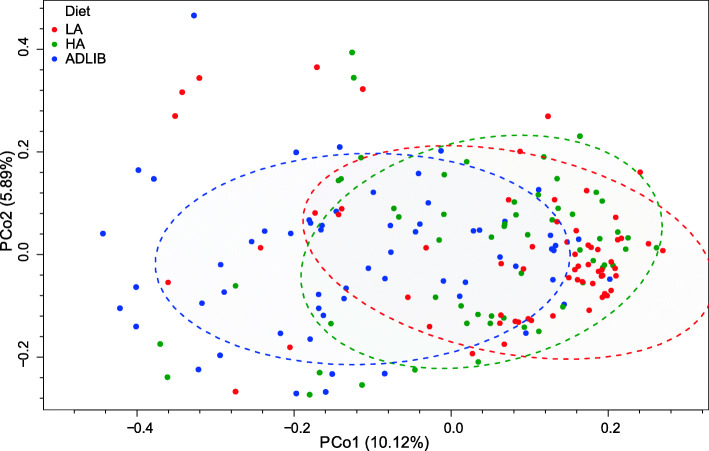
PCoA plot of faecal bacterial communities based on Bray-Curtis dissimilarities with datapoints coloured by treatment group, and treatment group confidence levels at 0.7 shown by ellipses

Principal Co-ordinates Analysis (PCoA) analyses based on Bray-Curtis dissimilarities of genus-level taxa were performed, and for each, permutation tests showed no significant difference (*P* > 0.05) in multivariate dispersions. Differences in community structures between the different feed treatments were significant (ANOSIM; *P* = 0.001; Fig. 3). The impact of source farm and sampling date was also examined. Source farm did not significantly affect the bacterial community structure (ANOSIM; *P* = 0.40, Fig. S1). However, despite the age of the calves being similar at the time of sampling, the sampling date was associated with community differences (ANOSIM; *P* = 0.001). As a prophylactic antibiotic treatment was administered to all calves between the fourth and fifth sampling dates, we examined the data to see whether this had a significant impact on the microbial community structures. The communities of samples collected before and after the antibiotics were administered were not significantly different (Fig. S1, ANOSIM; *P* = 0.40), however, differences by diet treatment remained significant in these two groups (ANOSIM, *P* = 0.001). Thus, differences in community structure due to sampling date may be largely due to uncontrolled environmental factors.

### Correlation analyses between faecal bacterial composition and fermentation products, and feed intake and calf performance

**Fig. 4:**
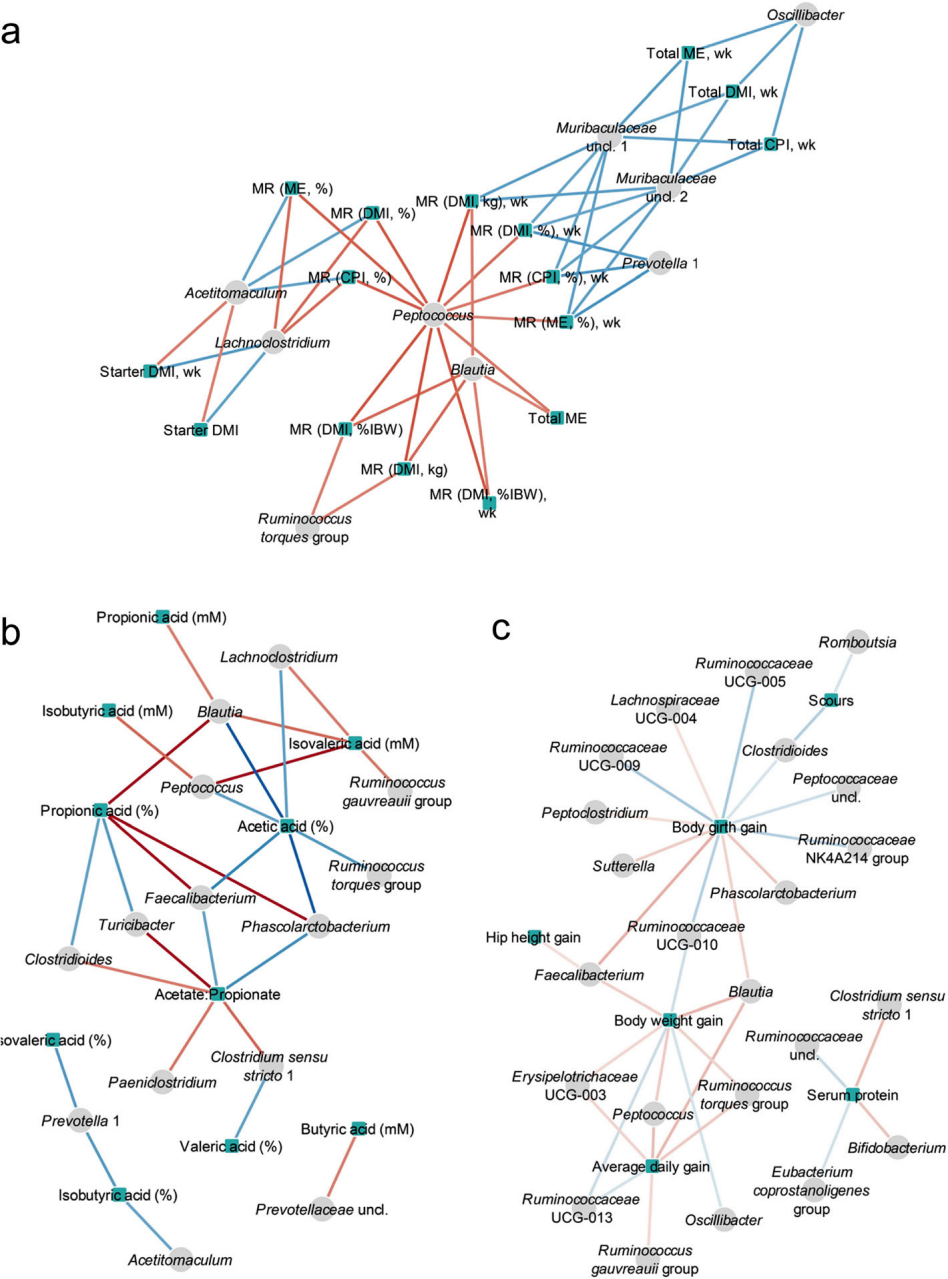
Network plots showing canonical correlations between bacterial community composition and (**a**) calf dietary intake data (Cor > |0.35|), (**b**) SCFA profiles (Cor > |0.5|), and (**c**) calf performance data (Cor > |0.3|). Abbreviations: MR, milk replacer; DMI, dry matter intake (kg); CPI, crude protein intake (kg); ME, metabolisable energy (MJ), %IBW, percentage of initial body weight; %, percentage of total intake by dry weight; wk., data obtained from the week prior to sampling only. Red and blue edges represent positive and negative correlations between nodes respectively, where colour intensity indicates the strength of correlation

Despite the calves being grouped by milk replacer allowance treatment, the actual intakes of milk replacer and calf starter feed varied widely among individuals within these groups, particularly for the ADLIB treatment (Fig. S2); and in general, calves that were offered more milk replacer consumed less starter [18]. To determine associations between the nutrient and energy intakes of the calves and their faecal bacterial communities, canonical correlation (Cor) analyses revealed moderate to high positive correlations (Cor > 0.50) between *Peptococcus, Blautia, Ruminococcus torques* group and total milk replacer dry matter intake (Fig. 4a). *Ruminococcus gauvreauii* group, *Lachnoclostridium, Subdoligranulum, Enterobacteriaceae* and *Faecalibacterium* exhibited moderate correlations (Cor 0.40–0.50) with total milk replacer dry matter intake (Fig. S3). When the proportions of dry matter, crude protein and metabolisable energy (ME) in the diet from milk replacer were considered, *Peptococcus* and *Lachnoclostrium* showed the greatest positive correlations (Cor > 0.53), while *Acetitomaculum* was most negatively correlated (Cor = − 0.49), instead showing greater abundances with increased starter intake (Fig. 4a). Correlations performed using daily average, or cumulative intake data over the duration of the calf trial, may not accurately reflect recent dietary intake levels, just prior to when the faecal samples were collected for microbiota assessment. Hence, we also compared intake data from the week just prior to sampling, and generally found similar relationships between the two intake measures with microbiota, and some stronger correlations evident (Fig. 4a and Fig. S3). Milk replacer intakes from the week just prior to faecal sampling correlated most strongly with the relative abundances of *Peptococcus*, *Tyzzerella* 4 and *Romboutsia* (Cor > 0.43), while the most negatively correlated taxa were *Prevotella* 1 and *Muribaculaceae* groups (Cor < − 0.50).

Comparison of the bacterial communities with SCFA profiles revealed a strong correlation between *Peptococcus* and isovaleric acid concentration (Cor = 0.60) (Fig. 4b) and *Faecalibacterium, Phascolarctobacterium* and *Blautia*, with the proportion of propionate produced (Cor > 0.60). *Turicibacter* was found to be negatively correlated with the proportion of propionate (Cor = − 0.53), and strong positively correlated with the acetate to propionate ratio (Cor = 0.63). Butyric acid concentrations and proportions showed moderate positive correlations with *Prevotella* 1 and uncultured members of *Prevotellaceae* (Cor > 0.47) (Fig. 4b and Fig. S4).

Calf growth and performance parameters were also compared to the microbial communities where general body weight and average daily gain correlated most strongly with *Peptococcus* and *Blautia* abundances (Cor > 0.35), and girth gain correlated most strongly with *Faecalibacterium* (Cor = 0.40) (Fig. 4c). Body girth was negatively correlated with members of the *Ruminococcaceae* (Cor < − 0.36) (Fig. 4c). The incidence and severity of scours was associated with *Sutterella* (Cor = 0.27) and *Rikenellaceae* (Cor = 0.22) (Fig. S5), but most negatively correlated with *Clostridioides* (Cor = − 0.37) (Fig. 4c). An interesting finding was a moderate correlation (Cor > 0.36) between *Clostridium* sensu stricto 1 and *Bifidobacterium* with total protein concentrations in serum at the onset of the trial. Serum total protein is a general indicator of colostrum intake in the first days of life and passive transfer of immune factors from colostrum to the calf and may also reflect time spent suckling from the dam.

The relationships between SCFA profiles and nutritional intakes (Fig. S6), and SCFA profiles and calf performance (Fig. S7) were also examined. Over the course of the trial, the intake of milk replacer (expressed as a percentage of calf initial body weight) correlated with the percentage of propionate produced (Cor = 0.55), while in general, the proportion of the diet coming from milk replacer was strongly correlated with proportion of propionate and BSCFA (Cor > 0.56) (Fig. S6). When intakes from the previous week only were considered, milk replacer intakes correlated most strongly with BSCFA concentrations (Cor > 0.67), but less so for proportion of propionate (Cor = 0.36). Of interest, neither the concentrations nor proportions of faecal butyrate correlated strongly with any nutritional intake measures (Fig. S6). The proportion of propionate displayed a moderately strong correlation with average daily gain (ADG) (Cor = 0.47), while the BSCFA concentrations both correlated most strongly with hip height gain (Cor > 0.44) (Fig. S7), but also with average daily and body weight gain.

## Discussion

Diet is a key contributor to intestinal microbiota diversity, and we here describe the faecal bacterial communities of nearly 200 calves with restricted to ad libitum access to a milk replacer-based diet. To our knowledge, this study represents one of the largest studies of the calf intestinal microbiota conducted to date and it has further provided insights into the relationships between gut microbiota with calf growth and health [18]. The faecal bacterial communities of the milk replacer-fed calves differed to those from our previous observations of 35-day old calves fed low and high allowances of whole milk. In the whole milk-fed calves, *Bacteroides* was the most abundant genus detected in the faeces of both treatments, comprising 13% of the 16S rRNA gene sequence reads, and *Faecalibacterium* was particularly prominent in the high milk allowance calves at ca. 7.7% (Moon et al., unpubl.). In contrast, the milk replacer-based diets supported communities that were dominated by *Ruminococcaceae* UCG_005 and *Lachnospiraceae* in the LA and HA treatments, followed by *Bacteroides*. Moreover, *Faecalibacterium* was much less abundant overall, averaging less than 1% in the LA group, to 2.6% in ADLIB group. The milk replacer used in this study is widely used on New Zealand farms and has a macronutrient profile that is similar to that of raw whole milk, being casein-based and containing dairy-derived fats. However, it is also supplemented with a coccidiostat and a mannan oligosaccharide (MOS)-based prebiotic derived from *Saccharomyces cerevisiae*, which are likely to influence the gut microbiota. Of the bacteria whose abundances were strongly promoted by milk replacer intake, *Romboutsia,* a member of the *Peptostreptococcaceae* [20, 21], was the most abundant, averaging 3.4% of 16S rRNA gene sequences in the ADLIB group compared to 2.0% in the LA group. The *Peptostreptococcaceae* were previously observed as being significantly more prominent in high protein compared to high carbohydrate diets in the cat faecal microbiota [22]. *Tyzzererella* 4 [23, 24] and *Peptococcus* abundances also correlated strongly with the proportion of milk replacer in the diet. Members of *Peptococcus* are able to use a variety of sulphur-containing compounds as terminal electron acceptors [25] and may play a greater role in sulphur metabolism, as well as protein metabolism, on diets with higher proportions of milk replacer, and therefore more protein. The age, stage of development, and greater proportion of solid feed in the diet are also likely to contribute to differences seen between the gut microbiota of the calves, though the influence of these is not clearly known.

The ADLIB diet treatment enhanced the faecal microbial community Shannon diversity index compared to the HA and LA treatments, whereas no differences in Chao1 index were observed between the treatment groups. This suggests that differences in the evenness of taxon abundances, rather than richness, were apparent between treatment groups, and may represent different impacts of the diet treatments on lowly abundant taxa. These findings may also reflect selection for greater functionality to utilise the higher proportion of protein and other milk replacer derived nutrients in the diet, compared to the more carbohydrate-rich diets of the calves on restricted milk allowances. Examination of the metagenomes from each of the diet treatments would provide further insights into the specific functions and pathways that are differentially represented among the different diet treatments.

Higher proportional intakes of milk replacer promoted faecal concentrations of the BSCFAs, isobutyrate and isovalerate, which are considered markers of protein fermentation and can be generated from the fermentation of branched-chain amino acids such as valine and leucine [26]. Their presence likely reflects higher concentrations of milk replacer-derived proteins and peptides arriving at the large intestine, where proteolytic fermentation takes place primarily in the distal as compared to the proximal colon [27, 28]. Protein fermentation in the gut is limited when carbohydrate is more readily available, such as in the proximal colon; and also, at lower pH values [26]. Previous studies report that the fermentation of branched-chain amino acids is mainly carried out by members of the genera *Clostridium, Peptostreptococcus* and *Bacteroides* [26], though these taxa did not correlate strongly with either milk replacer intakes or BSCFA concentrations in the present study. We did, however, observe positive correlations between BSCFA and *Blautia*, and BSCFA and *Peptococcus*, where species of the latter can produce isobutyric and isovaleric acids as major fermentation end-products [25]. The BSCFAs in the gut have been associated with changes in host lipid metabolism in adult humans [29], though an understanding of their role in the gut of the growing calf is limited. However, a recent study has shown that supplementation of BSCFAs in the total mixed ration of Holstein calves stimulated rumen metabolism and increased the ADG [30]. In the present study, higher milk replacer intakes as a proportion of initial body weight were associated with increased proportions of propionate in SCFA profiles, which may result in increased gluconeogenesis and contribute to higher ADG associated with ad libitum access to milk replacer [18]. Butyrate concentrations were of particular interest given their role in contributing to gut homeostasis and anti-inflammatory properties [31]. However, differences in butyrate concentration or proportion between treatment groups or with milk replacer intakes were not apparent. Despite this, butyrate concentrations were strongly negatively correlated with the incidence and severity of scours. This observation is although faecal SCFA were measured just prior to weaning, which took place several weeks after scours observations were made in the first few weeks of the trial [18]. Potentially, calves that did not contract scours, or had lower scours scores, naturally possessed a more butyrogenic microbiota that support better gut integrity. Alternatively, scours events may have impacted the microbiota’s capacity to produce butyrate or enhanced butyrate utilisation by the host, thus resulting in reduced concentrations in the faeces.

The gastrointestinal tracts of animals in utero are generally thought to be sterile and are rapidly colonised by microbes from the dam and the environment during and after birth [32]. The process of colonisation and development of the gut microbial community, and potential to manipulate to support beneficial microbiota is of great interest to understand factors that may impact the health and lifetime performance of the animal from birth. In this study, the abundances of *Clostridium* sensu stricto and *Bifidobacterium* correlated with total serum protein concentrations of the calves at the start of the trial, which is generally regarded to be indicative of colostrum intake in the first days of life, and potentially, the time spent in contact with the dam. *Bifidobacterium* is often among the first colonisers of gut environments, and in humans, its growth is supported by oligosaccharides abundant in breastmilk. While bovine milks contain less milk oligosaccharides than human breastmilk, bifidobacteria are detected in calves in the first few days of life [33] but thereafter, their abundance decreases. *Bifidobacterium* has been detected and isolated from bovine colostrum [34, 35], and may be an early coloniser of the gut through colostrum feeding. Indeed, evidence supports the notion that colostrum is a significant vector of microbiota-associated antimicrobial resistance genes, which displayed strong correlations between *E. coli* found in colostrum samples, and in the gut of the calves that fed it [35]. The MOS present in the milk replacer may further support *Bifidobacterium* growth, though its relative abundance in the faecal microbiota was low, did not differ significantly between diet treatments.

## Conclusions

Milk replacer allowances of pre-weaned calves impacted both faecal bacterial community composition and fermentation product profiles, and were associated with increased bacterial diversity and protein fermentation. Promotion of beneficial bacteria such as *Faecalibacterium* may contribute to hindgut development, energy harvest and growth. Moreover, a relationship between *Bifidobacterium* and initial serum protein levels was observed that suggests colostrum intake in the first days of life may have a lasting influence on the gut microbiota composition that can be detected just prior to weaning.

## Methods

### Experimental design and treatments

The calf trial, and all associated procedures for sampling and measurement taking, was approved by the AgResearch Grasslands Animal Ethics Committee, Palmerston North, New Zealand, application number 14249. Transportation of animals was conducted according to the Dairy Cattle Code of Welfare [36].

Full details of the calf trial have been described by Groenendijk et al. (2018) [18]. In brief, Kiwi cross (Holstein-Friesian x Jersey) calves born on Farmway Farm, Rongotea or Ohau Dairies, Horowhenua, New Zealand during the 2017 spring calving season were collected twice daily (e.g. 1–14 h after birth) from the calving paddocks. Calves received 2 L of first-milking colostrum at the time of collection (within 15 h of birth) and then 2 L twice daily until 2 days old. The calves were kept on their source farms for a minimum of 4 days old and were then transported to a dedicated calf-rearing facility at Farmway Farm.

All calves were manually fed whole milk using an artificial teat attached to a bottle (2 L in the morning and 2 L in the afternoon) from 3 to 7 days of age, and were trained to use automated milk feeders in stalls (CalfSMART, Palmerston North, New Zealand) twice daily from 8 to 10 days of age, fed 4 L/d milk replacer during this time.

Calves (*n* = 199) with no apparent sign of illness were allocated to the 3 treatments (LA (*n* = 67), HA (*n* = 65) and ADLIB (*n* = 66)), balanced for source farm, date of birth and body weight. A commercially available milk replacer (Ancalf, NZAgbiz Ltd., Hamilton, New Zealand) was diluted in lukewarm water (150 g/L) and fed to all calves using automated milk feeders (CalfSMART, Palmerston North, New Zealand). All calves had ad libitum access to a pelleted calf-starter (20% CP pellets, SealesWinslow Limited, Tauranga, New Zealand) by automated feeder, and clean drinking water. From the third week of the trial, calves were given ad libitum access to ryegrass hay. The nutritional composition for all feeds is provided in Table S3. Daily feed DM intake (milk replacer, calf starter and total) and nutrient intakes (ME and CP) were calculated for the pre-weaning period (d 0 to 83 on the study) for each calf from data collected on the automated feeders. Water and hay intakes were not measured.

All calves were vaccinated for prevention of leptospirosis and major clostridial diseases (Ultravac 7in1, Zoetis, Auckland, New Zealand) between 4 and 8 weeks of age, and all calves received antibiotics (Alamycin LA300, Norbrook, Auckland, New Zealand) on the same date as prevention for pneumonia. Calves were monitored and scored for the incidence of scours (faecal score of 2 or greater as described in the Calf Health Scorer criteria of the School of Veterinary Medicine, University of Wisconsin, Madison, USA) during the first 3 weeks on trial, after which, scouring was negligible. Calf body weight and dimension measurements were performed where body dimensions were taken of all calves at the start of the study and at weaning. Hip and wither height was determined using a measurement stick and heart girth measurement (the smallest circumference behind the forelegs) was taken using a measuring tape while the animal was standing on a flat surface and with head in an upright position. Blood serum protein levels were determined using the method described in Groenendijk et al. [18].

### Faecal sample collection and short chain fatty acid analysis

From days 67 ± 3 on trial, just prior to weaning, faecal samples were manually collected from calves and frozen on dry ice, then transported to the laboratory for storage at − 85 °C. Faecal samples were thawed on ice and used for SCFA analysis and DNA extraction. Approximately 1 g of material was weighed and diluted with 50% (v/w) phosphate buffered saline (137 mM NaCl, 2.7 mM KCl, 10 mM Na_2_HPO_4_ and 1.8 mM KH_2_PO_4_; pH 7.4), then centrifuged at 16,000 *g* for 10 min, 4 °C. A 270 μL aliquot of the supernatant was mixed with 30 μL of internal standard solution (20 mM 2-ethylbutyrate in 20% (v/v) phosphoric acid). Samples were frozen at − 20 °C, then thawed and centrifuged as above, prior to analysis by gas chromatography. Each supernatant sample (200 μL) was vigorously mixed with 100 μL concentrated HCl, and extracted twice with 800 μL diethyl ether for 1 min each. The supernatant extracts were pooled into a 2 ml vial and 800 μL of extract was derivatized with 100 μL of *N*-methyl-*N*-*t*-butyldimethylsilyltrifluoroacetamide (Sigma-Aldrich, St. Louis, MO, USA). The mixture was heated in a crimp top GC vial for 20 min at 80 °C and left for 48 h at room temperature to ensure complete derivitisation. Samples were analysed using a Shimadzu GC-2010 gas chromatograph (Shimadzu Corp., Kyoto, Japan) with a barium ionization detector 2010 (Shimadzu Corp.) and AOC 6000 autosampler (Shimadzu Corp.) and a Zebron ZB-5MS 30 m × 0.25 mm I.D. × 0.25 μm film capillary column (Phenomenex, Torrance CA, USA). Helium was used as carrier gas in conjunction with a He purifier (Valco Instruments Co. Inc., Houston TX, USA). Split injections (1 μL) were made with a ratio of 20:1 split, with column helium flow rate of 21.36 mL/min. Injector and detector temperatures were both 240 °C and column temperatures were programmed initially at 50 °C for 2 min, than increased to 130 °C with 5 °C per minute, followed by 15 °C per minute to 240 °C. The SCFA analyses were performed for every second animal when ranked by pre-weaning ADG within each treatment group, to ensure that the calves across the entire range of weight gain within each treatment group were represented in the subset of samples tested. For the LA, HA and ADLIB treatments, 33, 26 and 28 samples were analysed, respectively.

Total DNA was extracted from ca. 250 mg faecal sample using a commercial kit (Nucleospin Soil; Macherey-Nagel; Düren, Germany) according to the manufacturer’s instructions. To maximise DNA yield, a combination of buffer SL1 and enhancer solution SX was used, and physical disruption of the faecal sample was performed for 4 min at full speed using a Mini-Beadbeater-96 (Biospec Products, Bartlesville, USA). An addition DNA washing step was also included when the DNA was bound to the silica membrane. DNA was eluted with 50 μL elution buffer SE and stored at − 20 °C. All samples were further dialyzed for up to 4 h using 0.025 μm pore size membrane filters (MilliporeSigma, Burlington MA, USA) over double-distilled water. DNA concentration and purity was measured using an ND-1000 spectrophotometer (NanoDrop Technologies Inc., Wilmington DE, United States). Negative DNA extractions were not performed as the faecal samples are dense in microbial biomass, the risk of significant DNA contamination from the extraction kit was deemed neglible. Each batch of samples processed for DNA extractions and SCFA analyses was taken from across multiple sample collection dates, which minimised any potential confounding effect of collection date and processing date.

### 16S rRNA gene sequencing and analysis

The faecal bacterial communities were profiled by amplifying and sequencing the V4 region of the 16S rRNA gene for all DNA samples and negative controls (i.e. PCR reactions without DNA template), as previously described using the Illumina MiSeq platform (250-bp, paired end) [37]. In brief, the forward F515 primer which has an eight-nucleotide barcode unique to each sample and a two-nucleotide linker sequence (5′-NNNNNNNNGTGTGCCAGCMGCCGCGGTAA-3′) and the reverse R806 primer (5′-GGACTACHVGGGTWTCTAAT-3′) were used in the 16S rRNA gene amplification. PCR reactions were conducted in triplicate in 15 μL reactions containing 1× GoTaq Green Mastermix (Promega, Madison, WI, USA), 1 mM MgCl_2_ and 2 pmol of each primer. The PCR amplification conditions included an initial denaturation step of 2 min at 94 °C, followed by 25 cycles of 94 °C for 45 s, 50 °C for 60 s, and 72 °C for 90 s, followed by a final extension step at 72 °C for 10 min. Triplicate reactions were subsequently combined and purified using a PCR purification column (QIAGEN, Hilden, Germany) and submitted to the DNA Technologies & Expression Analysis Core at UC Davis for sequencing on an Illumina MiSeq platform. Sequence data were quality filtered as previously described [37] and loaded into QIIME2 (version 2019.1) [38] using the default workflow. Briefly, reads were demultiplexed using barcode sequence associated with individual sample using Sabre software (*sabre pe*) (https://github.com/najoshi/sabre). Demultiplexed sequences were then processed through the DADA2 pipeline for quality control and the feature table was constructed at amplicon sequence variant (*ASV*) level. Eight samples (2 LA, 3 HA, 3 ADLIB) had < 3000 reads and were excluded from the feature table and further analysis. The sequence negative control samples averaged 133 reads per sample and were also omitted from further analysis. A naïve Bayesian pre-trained classifier for the V3-V4 region of 16S rRNA gene using the Silva 132 99% OTUs from 515F/806R region of sequences’ database was used for assigning taxonomic classifications to ASVs. The taxonomic composition of the samples was then summarised using the associated metadata. Bacterial data were retained after filtering non-bacterial taxa (e.g. eukaryotic and archaeal reads). Genus-level read abundances were analysed for alpha diversity using Shannon diversity and Chao1 index metrics with subsampling at 3000 reads per sample, and with the mean of 10 iterations used for diversity values. Data were converted into bacterial relative abundances for downstream statistical analyses.

### Statistical analyses

The bacterial community composition data were analysed in R version 3.6.1 [39] implemented in RStudio V1.2.1335 [40]. PCoA on Bray-Curtis dissimilarity matrices [41] and permutation tests for homogeneity of multivariate dispersions (using 999 permutations) were conducted using the VEGAN R package [42]. Analysis of similarities (ANOSIM) [43] was also performed using VEGAN R package [42]. Permutational multivariate analysis of variance (PERMANOVA) was performed using base functions in R and then PERMANOVA. Least significant difference (LSD) post hoc analysis were performed using R package, agricolae [44]. The R package rstatix (https://rpkgs.datanovia.com/rstatix/) was used to perform Kruskal-Wallis rank sum and Wilcoxon rank sum tests. Canonical correlation analysis was performed using the MixOmics R package [45] to correlate microbiome community structures with parameters associated with calf growth and performance [18], and faecal SCFA data. Heatmaps were generated in R. Correlation network data was visualized using the igraph package for R [46] and Cytoscape V3.5.1 [47].

## Supplementary Information

**Additional file 1: Table S1**. Relative abundances (%) of bacterial family-level taxa by diet group. **Table S2**. Relative abundances (%) of bacterial genus-level taxa by diet group.

**Additional file 2: Fig. S1**. PCoA of calf faecal bacterial communities in relation to farm source and sampling date. **Fig. S2**. Variation in calf milk replacer intakes. **Fig. S3**. Correlation heatmap between bacterial community composition and calf dietary intakes. **Fig. S4**. Correlation heatmap between bacterial community composition and SCFA profiles. **Fig. S5**. Correlation heatmap between bacterial community composition and calf performance data. **Fig. S6**. Correlation heatmap between SCFA profiles and calf dietary intakes. **Fig. S7**. Correlation heatmap between SCFA profiles and calf performance data. **Table S3**. Nutritional composition of milk replacer, pelleted calf starter and ryegrass hay.

## Data Availability

The sequence datasets supporting the conclusions of this article are available in the NCBI sequence read archive (https://www.ncbi.nlm.nih.gov/sra), BioProject PRJNA650595; and all data and metadata files used for statistical analysis are available in GitHub (https://github.com/kusandeep/Calf-microbiome), together with the R Markdown file.

## Abbreviations

ADLIB: Ad libitum (milk allowance)
ADG: Average daily gain
ANOSIM: Analysis of similarities
ANOVA: Analysis of variance
ASV: Amplicon sequence variant
BSCFA: Branched short chain fatty acid
CP: Crude protein
CPI: Crude protein intake
DM: Dry matter
DMI: Dry matter intake
HA: High allowance (of milk replacer)
LA: Low allowance (of milk replacer)
ME: Metabolisable energy
MR: Milk replacer
NEFA: Non-esterified fatty acids
PCoA: Principal co-ordinates analysis
SCFA: Short chain fatty acid

## References

[1] Ørskov ER (1972). Reflex closure of the oesophageal groove and its potential application in ruminant nutrition. S Afr J Anim Sci.

[2] Khan MA, Bach A, Weary DM, von Keyserlingk MAG (2016). Invited review: transitioning from milk to solid feed in dairy heifers. J Dairy Sci.

[3] Youngblut ND, Reischer GH, Walters W, Schuster N, Walzer C, Stalder G, Ley RE, Farnleitner AH (2019). Host diet and evolutionary history explain different aspects of gut microbiome diversity among vertebrate clades. Nat Commun.

[4] Dill-McFarland KA, Weimer PJ, Breaker JD, Suen G (2019). Diet influences early microbiota development in dairy calves without long-term impacts on milk production. Appl Environ Microbiol.

[5] Oikonomou G, Teixeira AGV, Foditsch C, Bicalho ML, Machado VS, Bicalho RC (2013). Fecal microbial diversity in pre-weaned dairy calves as described by pyrosequencing of metagenomic 16S rDNA. Associations of *Faecalibacterium* species with health and growth. PLoS One.

[6] Uyeno Y, Sekiguchi Y, Kamagata Y (2010). rRNA-based analysis to monitor succession of faecal bacterial communities in Holstein calves. Lett Appl Microbiol.

[7] Song Y, Malmuthuge N, Steele MA, Guan LL (2018). Shift of hindgut microbiota and microbial short chain fatty acids profiles in dairy calves from birth to pre-weaning. FEMS Microbiol Ecol.

[8] Dias J, Marcondes MI, Motta de Souza S, da Mata ESB C, Fontes Noronha M, Tassinari Resende R (2018). Bacterial community dynamics across the gastrointestinal tracts of dairy calves during preweaning development. Appl Env Microbiol.

[9] Malmuthuge N, Griebel PJ (2014). L. GL. Taxonomic identification of commensal bacteria associated with the mucosa and digesta throughout the gastrointestinal tracts of preweaned calves. Appl Env Microbiol.

[10] Malmuthuge N, Li M, Goonewardene LA, Oba M, Guan LL (2013). Effect of calf starter feeding on gut microbial diversity and expression of genes involved in host immune responses and tight junctions in dairy calves during weaning transition. J Dairy Sci.

[11] Ley RE, Hamady M, Lozupone C, Turnbaugh P, Ramey RR, Bircher JS (2008). Evolution of mammals and their gut microbes. Science.

[12] Foditsch C, Pereira RVV, Ganda EK, Gomez MS, Marques EC, Santin T (2016). Oral administration of *Faecalibacterium prausnitzii* decreased the incidence of severe diarrhea and related mortality rate and increased weight gain in preweaned dairy heifers. PLoS One.

[13] Dias NW, Timlin CL, Santili FV, Wilson TB, White RR, Mercadante VRG (2018). Establishing the efficacy of *Faecalibacterium prausnitzii* as a probiotic to enhance pre-weaning health, growth and performance of beef calves. J Anim Sci.

[14] Soberon F, Raffrenato E, Everett RW, Van Amburgh ME (2012). Preweaning milk replacer intake and effects on long-term productivity of dairy calves. J Dairy Sci.

[15] Geiger AJ, Parsons CLM, James RE, Akers RM (2016). Growth, intake, and health of Holstein heifer calves fed an enhanced preweaning diet with or without postweaning exogenous estrogen. J Dairy Sci.

[16] Khan MA, Weary DM, von Keyserlingk MAG (2011). Hay intake improves performance and rumen development of calves fed higher quantities of milk. J Dairy Sci.

[17] Steele MA, Penner GB, Chaucheyras-Durand F, Guan LL (2016). Development and physiology of the rumen and the lower gut: targets for improving gut health. J Dairy Sci.

[18] Groenendijk M, Lowe K, Schreurs N, Molenaar A, McCoard SA, Luo D (2018). Growth performance of crossbred dairy calves fed different milk allowances using an automatic feeding system. NZ J Anim Sci Prod.

[19] Quast C, Pruesse E, Yilmaz P, Gerken J, Schweer T, Yarza P, Peplies J, Glöckner FO (2013). The SILVA ribosomal RNA gene database project: improved data processing and web-based tools. Nucleic Acids Res.

[20] Gerritsen J, Fuentes S, Grievink W, van Niftrik L, Tindall BJ, Timmerman HM, Rijkers GT, Smidt H (2014). Characterization of *Romboutsia ilealis* gen. Nov., sp. nov., isolated from the gastro-intestinal tract of a rat, and proposal for the reclassification of five closely related members of the genus *Clostridium* into the genera *Romboutsia* gen. Nov., *Intestinibacter* gen. Nov., *Terrisporobacter* gen. Nov. and *Asaccharospora* gen. Nov. Int J Syst Evol Microbiol.

[21] Gerritsen J, Umanets A, Staneva I, Hornung B, Ritari J, Paulin L, Rijkers GT, de Vos WM, Smidt H (2018). *Romboutsia hominis* sp. nov., the first human gut-derived representative of the genus *Romboutsia*, isolated from ileostoma effluent. Int J Syst Evol Microbiol.

[22] Bermingham EN, Young W, Butowski CF, Moon CD, Maclean PH, Rosendale D (2018). The fecal microbiota in the domestic cat (Felis catus) is influenced by interactions between age and diet; A five year longitudinal study. Front Microbiol.

[23] Holdeman LV, Moore WEC (1974). New genus, *Coprococcus*, twelve new species, and emended descriptions of four previously described species of bacteria from human feces. Int J Syst Evol Microbiol.

[24] Yutin N, Galperin MY (2013). A genomic update on clostridial phylogeny: gram-negative spore formers and other misplaced clostridia. Environ Microbiol.

[25] Shkoporov AN, Efimov BA, Kondova I, Ouwerling B, Chaplin AV, Shcherbakova VA, Langermans JAM (2016). *Peptococcus simiae* sp. nov., isolated from rhesus macaque faeces and emended description of the genus *Peptococcus*. Int J Syst Evol Microbiol.

[26] Smith EA, Macfarlane GT (1998). Enumeration of amino acid fermenting bacteria in the human large intestine: effects of pH and starch on peptide metabolism and dissimilation of amino acids. FEMS Microbiol Ecol.

[27] Macfarlane GT, Gibson GR, Beatty E, Cummings JH (1992). Estimation of short-chain fatty acid production from protein by human intestinal bacteria based on branched-chain fatty acid measurements. FEMS Microbiol Ecol.

[28] Macfarlane GT, Gibson GR, Cummings JH (1992). Comparison of fermentation reactions in different regions of the human colon. J Appl Bacteriol.

[29] Granado-Serrano AB, Martín-Garí M, Sánchez V, Riart Solans M, Berdún R, Ludwig IA, Rubió L, Vilaprinyó E, Portero-Otín M, Serrano JCE (2019). Faecal bacterial and short-chain fatty acids signature in hypercholesterolemia. Sci Rep.

[30] Liu YR, Du HS, Wu ZZ, Wang C, Liu Q, Guo G (2020). Branched-chain volatile fatty acids and folic acid accelerated the growth of Holstein dairy calves by stimulating nutrient digestion and rumen metabolism. Animal.

[31] Bedford A, Gong J (2018). Implications of butyrate and its derivatives for gut health and animal production. Anim Nutr.

[32] Yañez-Ruiz DR, Abecia L, Newbold CJ (2015). Manipulating rumen microbiome and fermentation through interventions during early life: a review. Front Microbiol.

[33] Malmuthuge N, Griebel PJ, Guan Le L (2015). The gut microbiome and its potential role in the development and function of newborn calf gastrointestinal tract. Front Vet Sci.

[34] De Dea LJ, Santarelli M, Yamaguishi CT, Soccol CR, Neviani E (2011). Recovery and identification of bovine colostrum microflora using traditional and molecular approaches. Food Technol Biotech.

[35] Liu J, Taft DH, Maldonado-Gomez MX, Johnson D, Treiber ML, Lemay DG, DePeters EJ, Mills DA (2019). The fecal resistome of dairy cattle is associated with diet during nursing. Nat Commun.

[36] National Animal Welfare Advisory Committee: Code of Welfare: Dairy Cattle. In. Edited by Welfare MfPIMRABA. Wellington, New Zealand; 2016.

[37] Liu J, Zhu Y, Jay-Russell M, Lemay DG, Mills DA (2020). Reservoirs of antimicrobial resistance genes in retail raw milk. Microbiome.

[38] Bolyen E, Rideout JR, Dillon MR, Bokulich NA, Abnet CC, Al-Ghalith GA (2019). Reproducible, interactive, scalable and extensible microbiome data science using QIIME 2. Nat Biotechnol.

[39] Team RC (2018). R: a language and environment for statistical computing. In.

[40] Team R (2020). RStudio: Integrated Development for R.

[41] Beals EW. Bray-Curtis ordination: An effective strategy for analysis of multivariate ecological data. In: MacFadyen A, Ford ED, editors. Advances in Ecological Research. London: Academic Press; 1984. p. 1–55.

[42] Dixon P (2003). VEGAN, a package of R functions for community ecology. J Veg Sci.

[43] Clarke KR (1993). Non-parametric multivariate analyses of changes in community structure. Aust J Ecol.

[44] de Mendiburu F: Statistical procedures for agricultural research using R. In., 1.3–1 edn; 2019.

[45] Rohart F, Gautier B, Singh A, Lê Cao K-A (2017). MixOmics: An R package for ‘omics feature selection and multiple data integration. PLoS Comput Biol.

[46] Csardi G, Nepusz T. The igraph software package for complex network research. InterJournal. 2006 Complex Systems:1695.

[47] Shannon P, Markiel A, Ozier O, Baliga NS, Wang JT, Ramage D, Amin N, Schwikowski B, Ideker T (2003). Cytoscape: a software environment for integrated models of biomolecular interaction networks. Genome Res.

