# Supplementary material for: Effect of milk replacer allowance on calf faecal bacterial community profiles and fermentation
Source: Anim Microbiome. 2021 Apr 1;3:27. doi: 10.1186/s42523-021-00088-2 (PMC8017768; doi:10.1186/s42523-021-00088-2)
Supplement: Supplementary file 2 — Additional file 2: Fig. S1. PCoA of calf faecal bacterial communities in relation to farm source and sampling date. Fig. S2. Variation in calf milk replacer intakes. Fig. S3. Correlation heatmap between bacterial community composition and calf dietary intakes. Fig. S4. Correlation heatmap between bacterial community composition and SCFA profiles. Fig. S5. Correlation heatmap between bacterial community composition and calf performance data. Fig. S6. Correlation heatmap between SCFA profiles and calf dietary intakes. Fig. S7. Correlation heatmap between SCFA profiles and calf performance data. Table S3. Nutritional composition of milk replacer, pelleted calf starter and ryegrass hay. [file 42523_2021_88_MOESM2_ESM.pdf]

Effect of milk replacer allowance on calf faecal bacterial community profiles and fermentation

Sandeep Kumar[1], M. Ajmal Khan[1], Emma Beijer[2], Jinxin Liu[3,4], Katherine K. Lowe[1], Wayne Young[1], David A. Mills[3,4,5], Christina D. Moon[1*]

[1]AgResearch Limited, Grasslands Research Centre, Palmerston North, New Zealand

[2]Animal Nutrition Group, Wageningen University and Research, Wageningen, The Netherlands

[3]Department of Food Science and Technology, Robert Mondavi Institute for Wine and Food Science, University of California, Davis, One Shields Ave., Davis, CA 95616, USA

[4]Foods for Health Institute, University of California, Davis, California, One Shields Ave., Davis, CA 95616, USA

[5]Department of Viticulture and Enology, Robert Mondavi Institute for Wine and Food Science, University of California, Davis, California, One Shields Ave., Davis, CA 95616, USA

*Correspondence: christina.moon@agresearch.co.nz

Supplementary Figures

Figure S1. PCoA of calf faecal bacterial communities in relation to farm source and sampling date

PCoA plots based on Bray-Curtis dissimilarities are shown with data are coloured by (**a**) calf farm source, (**b**) sampling date, where dates pre- and post-antibiotic administration are denoted by shades of blue and red, respectively.

a

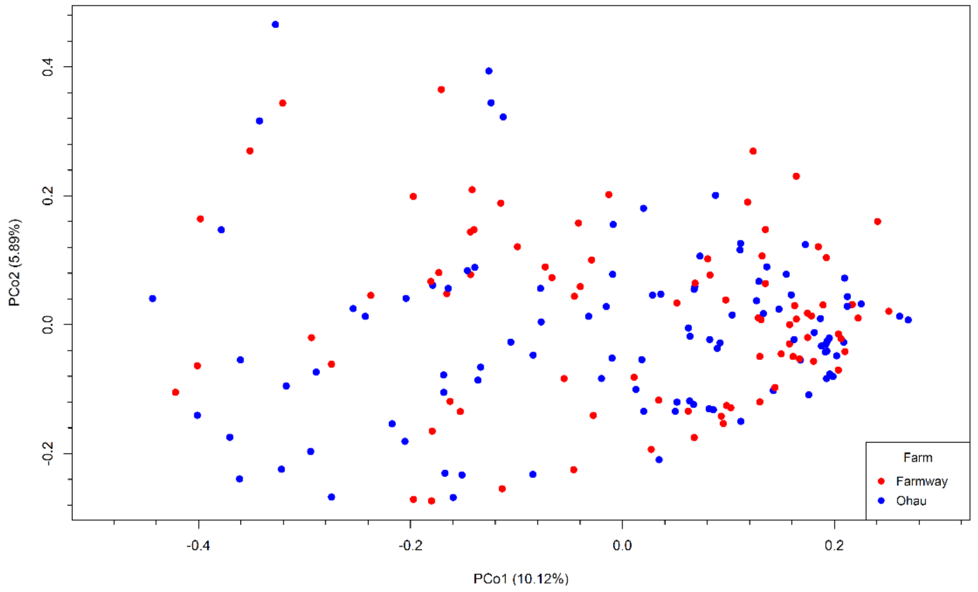

b

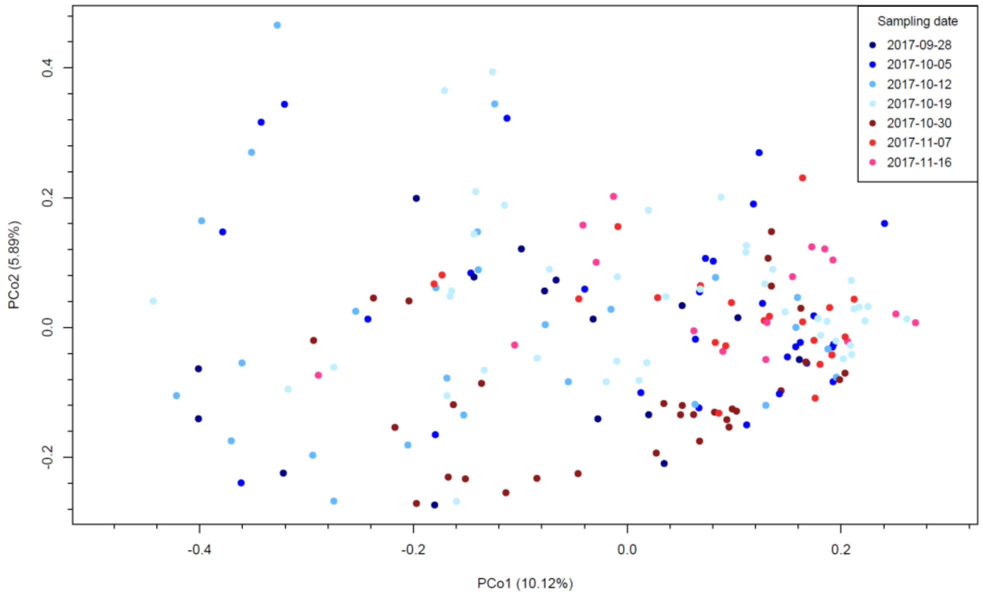

Figure S2. Variation in calf milk replacer intakes

Box plots overlaid with datapoints showing the spread of average daily milk replacer (MR) intakes, as dry matter (DM) intake as a percentage of the initial bodyweight (BW) for each of the treatment groups, LA, HA and ADLIB.

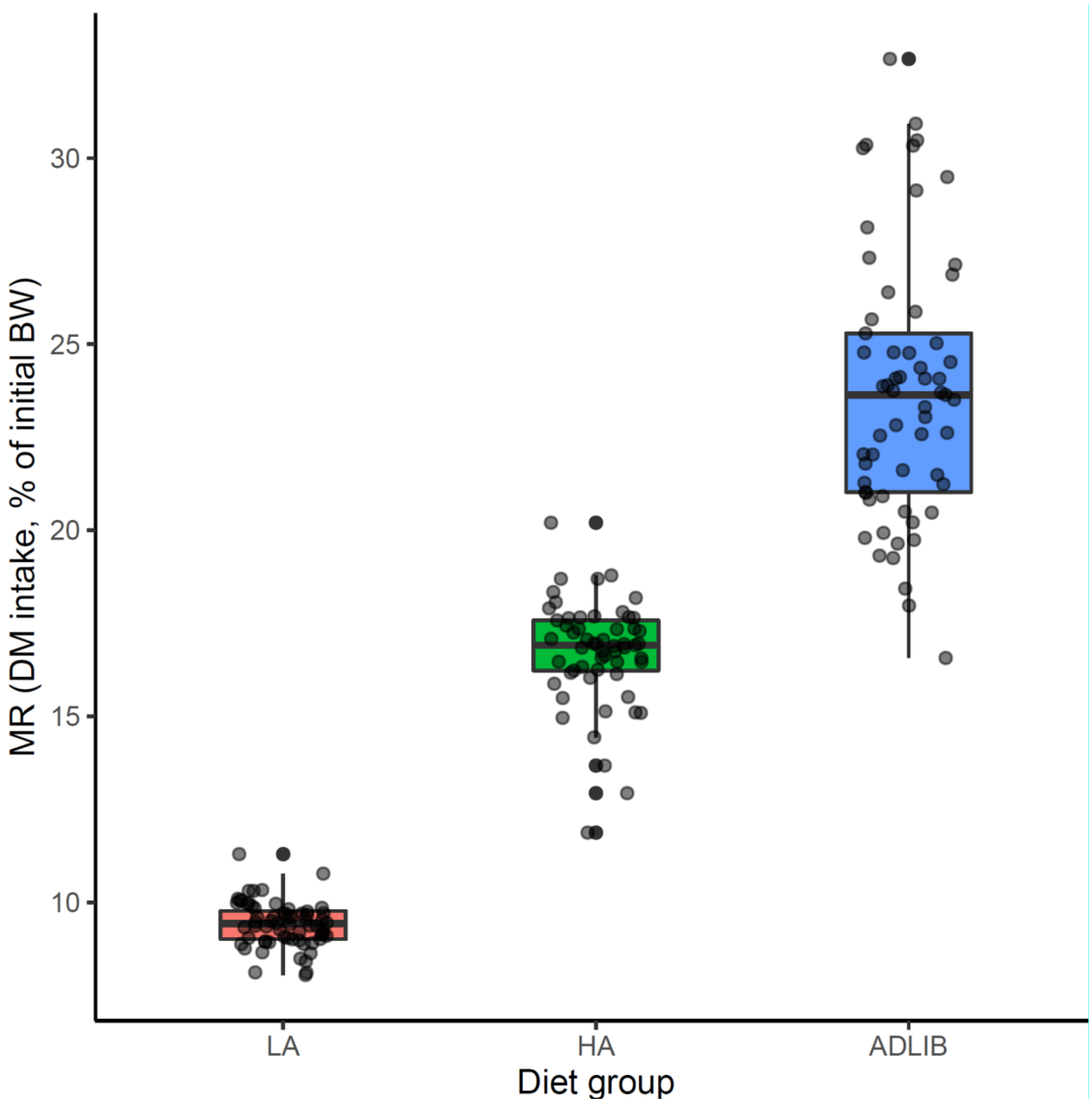

Figure S3. Correlation heatmap between bacterial community composition and calf dietary intakes

Heatmap depicting canonical correlations (Cor, maximum Cor >|0.3|) between calf faecal bacterial community composition and dietary intakes in the week prior to sampling (wk) and total intakes during the trial until sampling. Abbreviations: MR, milk replacer; DM, dry matter; CP, crude protein; ME, metabolisable energy. Green scale bars depict bacterial relative abundance.

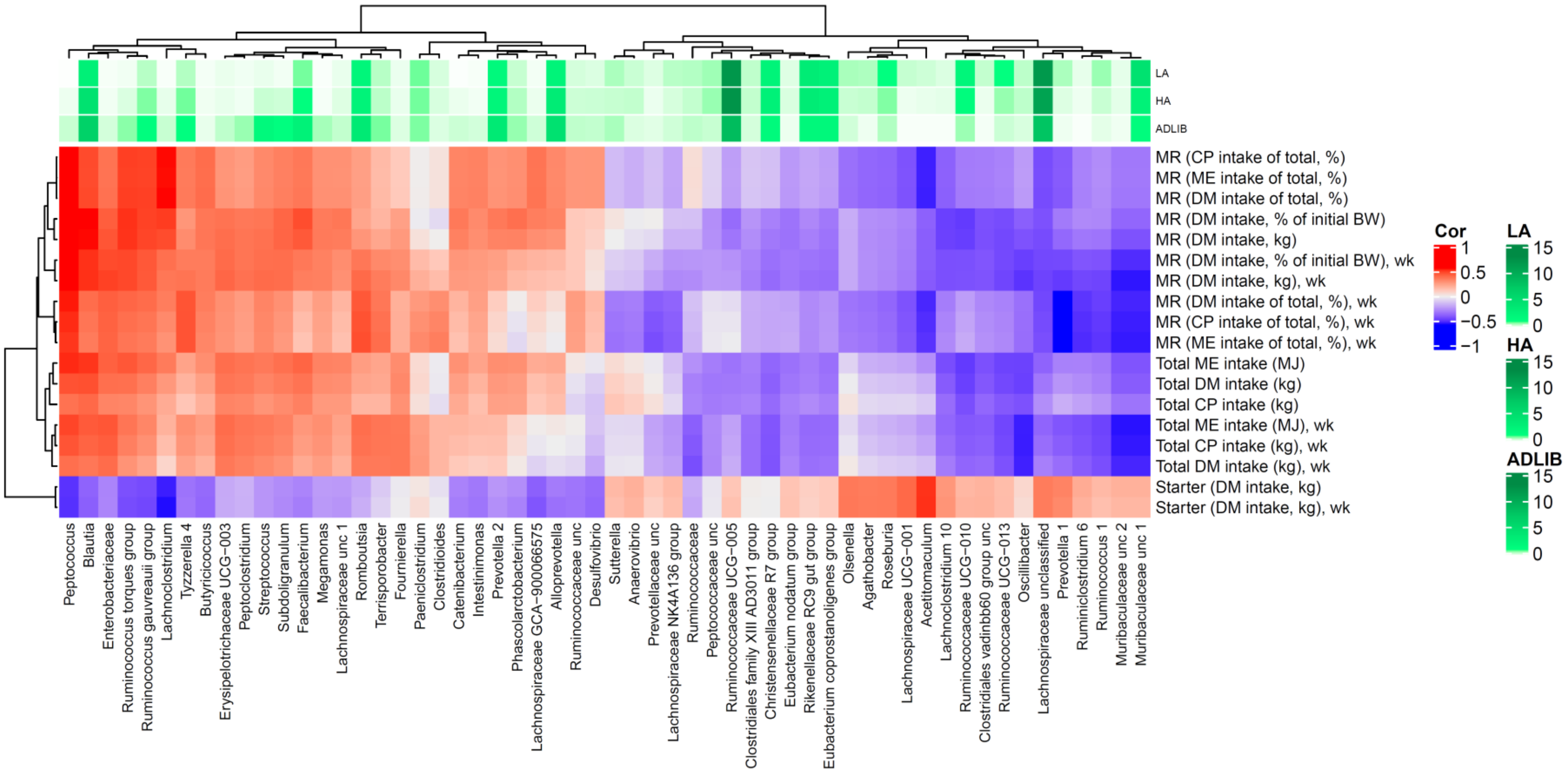

Figure S4. Correlation heatmap between bacterial community composition and SCFA profiles

Heatmap depicting canonical correlations (Cor, maximum Cor >|0.1|) between calf faecal bacterial community composition and SCFA concentrations (mM) and as a proportion of total SCFA concentration (%). Green scale bars depict bacterial relative abundance.

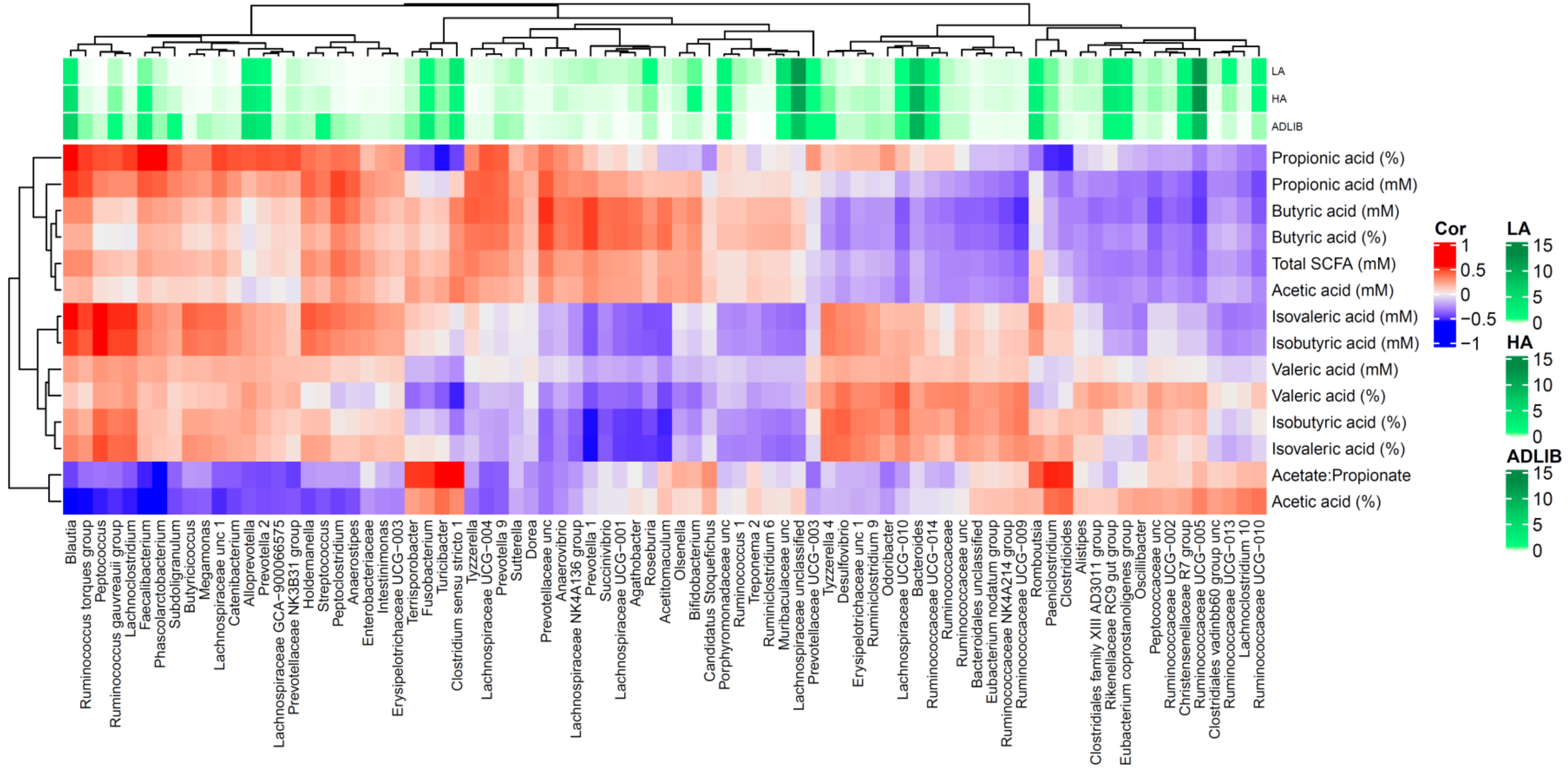

Figure S5. Correlation heatmap between bacterial community composition and calf performance data

Heatmap depicting canonical correlations (Cor, maximum Cor >|0.25|) between calf faecal bacterial community composition and calf growth, total serum protein and scours scores. Green scale bars depict bacterial relative abundance.

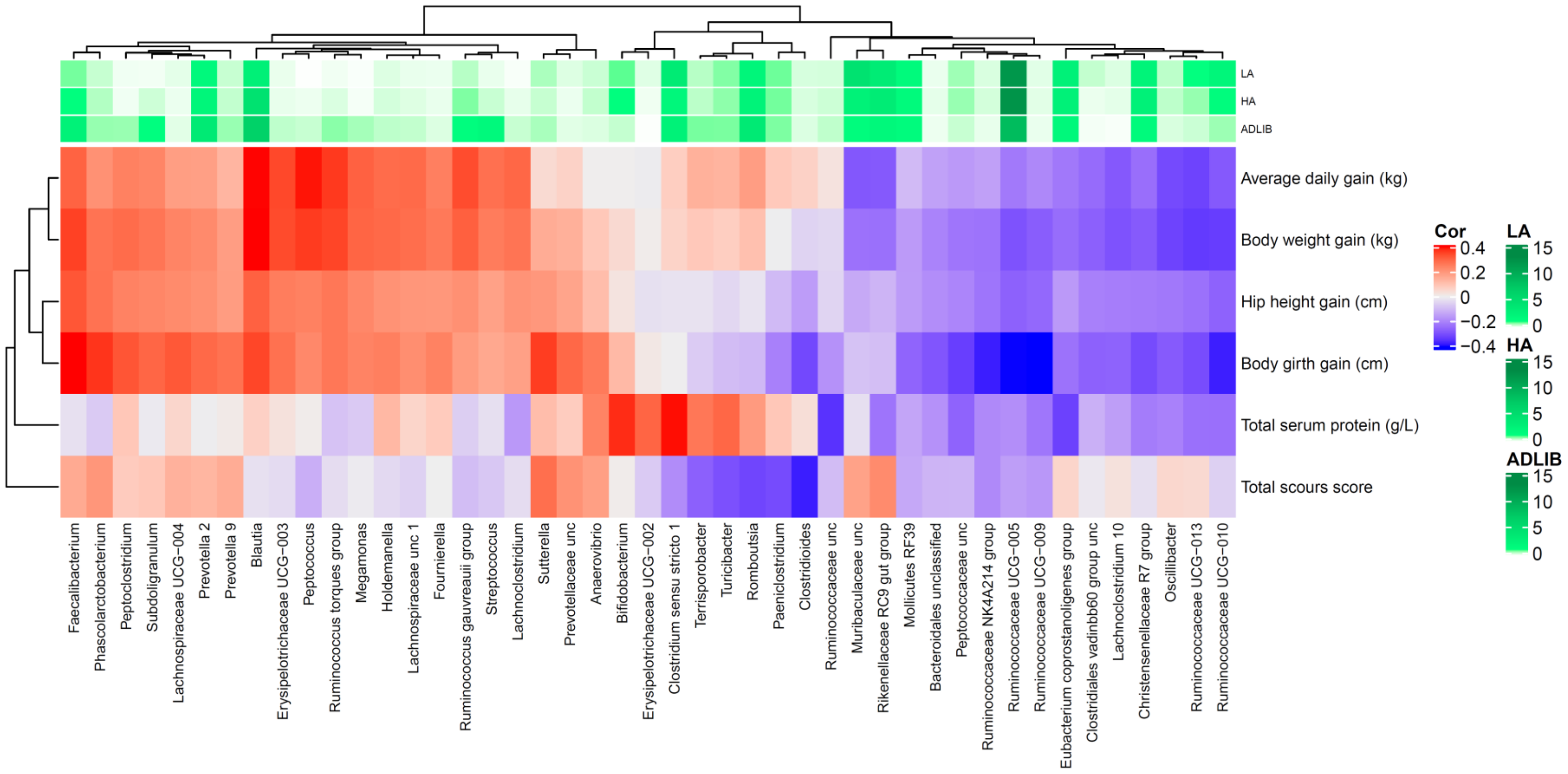

Figure S6. Correlation heatmap between SCFA profiles and calf dietary intakes

Heatmap depicting canonical correlations (top left) between SCFA measurements and dietary intakes for variables with maximum correlations >|0.3|.

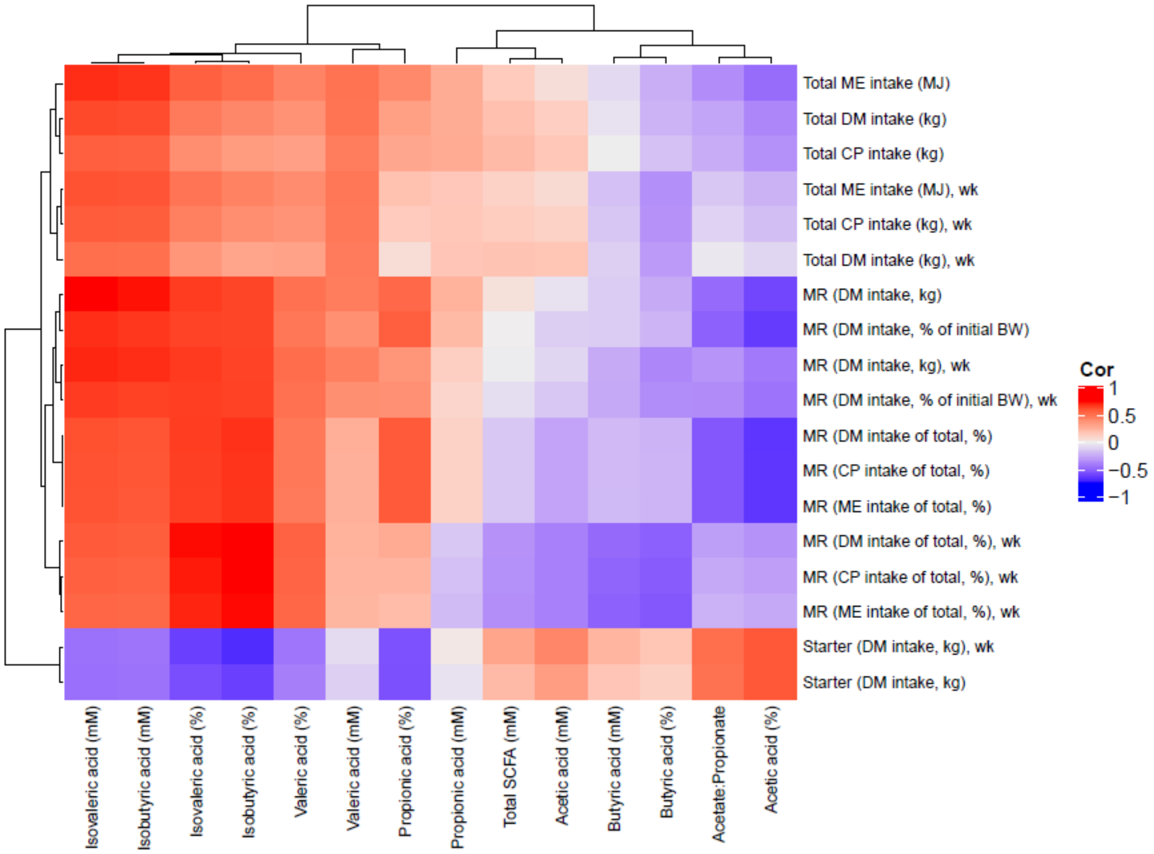

Figure S7. Correlation heatmap between SCFA profiles and calf performance data

Heatmap depicting canonical correlations (top left) between SCFA measurements and calf performance for variables with maximum correlations >|0.3|.

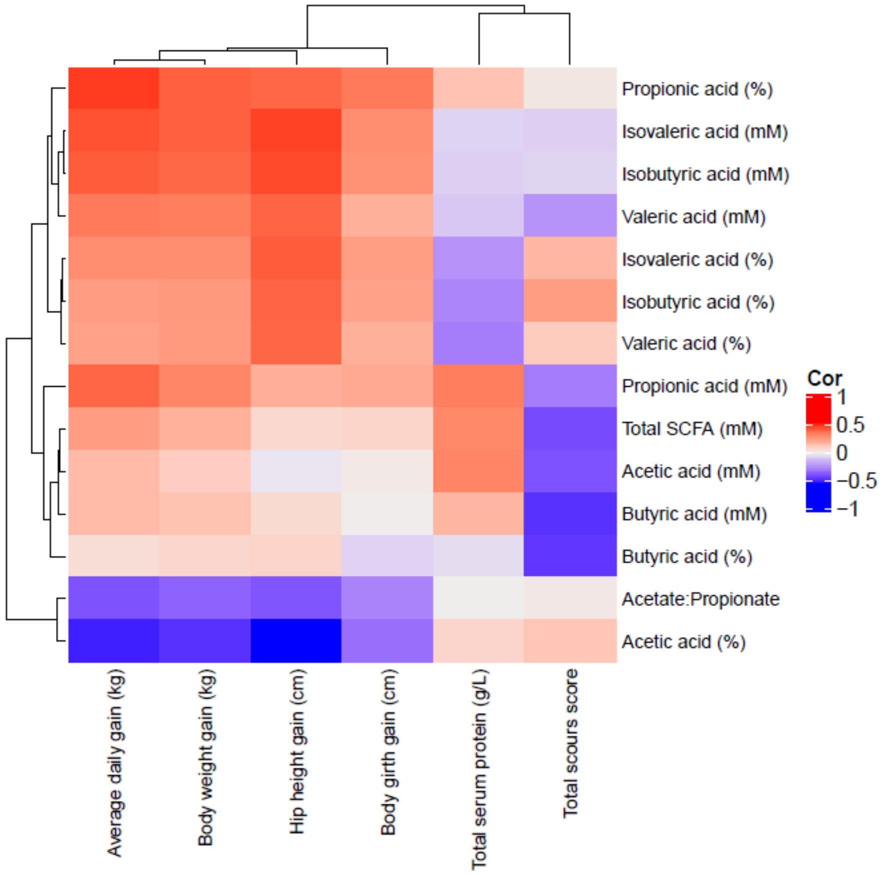

Supplementary Tables

Table S1. Relative abundances of bacterial taxa by family

See Excel file for data

Table S2. Relative abundances of bacterial taxa by genus

See Excel file for data

Table S3. Nutritional composition of milk replacer, pelleted calf starter and ryegrass hay

Analysis, DM basis[1]	Calf starter[2]	Milk replacer[3]	Hay
DM, %	87.1	96.1	92.6
ME, Mcal/kg DM[4]	3.27	4.92	2.63
CP, %	20.4	24.9	12.7
Lactose, %	-	39.5	
ADF, %	4.1	-	36.6
NDF, %	9.6	-	47.7
Ash, %	7.5	6.4	10.0
OM, %	92.5	-	-
Soluble sugars, %	5.55	-	-
Starch, %	40.5	-	-
Crude Fat, %	2.05	21.0	-
NSC, %	60.4	-	-
OM digestibility in-vitro, %	93.1	-	68.9
pH	-	-	4.1

[1]except where otherwise stated.

[2]20% pellets, SealesWinslow Ltd., Tauranga, New Zealand.

[3]Ancalf, NZAgbiz Ltd., Hamilton, New Zealand.

[4]ME in calf starter was calculated according to the AFRC Technical Committee on Responses to Nutrients (1993). Energy and protein requirements of ruminants, CAB International, Wallingford, UK; and in MR according to equations in the 7th edition of Nutrient Requirements of Dairy Cattle (2001). National Academies Press, Washington DC, USA.
